# Search, Memory, and Choice Error: An Experiment

**DOI:** 10.1371/journal.pone.0126508

**Published:** 2015-06-29

**Authors:** Adam Sanjurjo

**Affiliations:** Fundamentos del Análisis Económico, Universidad de Alicante, Alicante, Spain; Middlesex University London, UNITED KINGDOM

## Abstract

Multiple attribute search is a central feature of economic life: we consider much more than price when purchasing a home, and more than wage when choosing a job. An experiment is conducted in order to explore the effects of cognitive limitations on choice in these rich settings, in accordance with the predictions of a new model of search memory load. In each task, subjects are made to search the *same* information in one of two orders, which differ in predicted memory load. Despite standard models of choice treating such variations in order of acquisition as irrelevant, lower predicted memory load search orders are found to lead to substantially fewer choice errors. An implication of the result for search behavior, more generally, is that in order to reduce memory load (thus choice error) a limited memory searcher ought to deviate from the search path of an unlimited memory searcher in predictable ways-a mechanism that can explain the systematic deviations from optimal sequential search that have recently been discovered in peoples' behavior. Further, as cognitive load is induced endogenously (within the task), and found to affect choice behavior, this result contributes to the cognitive load literature (in which load is induced exogenously), as well as the cognitive ability literature (in which cognitive ability is measured in a separate task). In addition, while the information overload literature has focused on the detrimental effects of the quantity of information on choice, this result suggests that, holding quantity constant, the order that information is observed in is an essential determinant of choice failure.

## Introduction

Multiple attribute search is a central feature of economic life: we consider much more than wage when choosing a job, and much more than price when purchasing a home. Accordingly, an abundance of popular consumer websites facilitate the comparison of multiple alternatives across multiple attributes, for a wide variety of products, from digital cameras to cars and homes, to health care plans and 401k’s [1, 2].

Nevertheless, while optimal search policies have been fully characterized for a wide variety of search tasks in which a single attribute of an alternative, usually its price, determines its desirability, with varying assumptions about sampling opportunities, value distributions, search costs, number of searchable alternatives, and recall options [3–6], relatively little is known about optimal search in multiple attribute problems, particularly as they begin to more closely resemble those rich real world environments in which up to a large number of alternatives and attributes can be searched in any order. See S5 Appendix for a brief review of the literature.

In spite of the theoretical results being limited, an extensive experimental literature studies multiple attribute search behavior in problems that are relatively large dimensionally, and place relatively few restrictions on search behavior (see, for example, [1, 2, 7–13]). Such experiments typically track, not only subjects’ choice of alternative, but also their entire sequence of information search—either with eye-tracking (e.g. [14, 15]), or a mechanical analog such as Mouselab, in which subjects “click” open closed boxes in order to observe information (e.g. [16, 17]). An experimental design of particular interest is that of [8] (henceforth GLMW), which records the search behavior of 390 subjects in problems with a large number of alternatives (eight) and attributes (ten), full recall (meaning that any alternative can be chosen following any history of search), no order restrictions (meaning that any attribute of any alternative can be searched at any time), objectively correct choices, and sufficient structure for one to at least attempt to study optimal sequential search (i.e. in GLMW’s task attribute values are numerical, and subjects are fully informed about their distributions and relative information values). Nevertheless, although optimal sequential search would be a natural benchmark with which to compare subjects’ behavior, GLMW find that the high dimensionality of their problem makes the optimal policy both analytically and numerically intractable, so they instead test several behavioral models.

Despite the intractability of the optimal policy, a partial characterization is later introduced in [18], and when applied to GLMW’s data subjects are found to systematically (and substantially) deviate from optimality by searching too deeply within alternatives (at the expense of searching other alternatives), and switching too adjacently between alternatives (how initially “promising” each alternative is to search is transparent, and is independent of its spatial position). These costly deviations cannot be explained by standard candidate explanations (such as myopia and costly re-optimization [18]). By contrast, a new model of memory load in search [19], inspired by models from the psychology literature in which the decision process is decomposed into “elementary information processes,” as in the original work of [20, 21], and later “effort-accuracy” models [7, 22–27], reveals that these systematic deviations—as well as a variety of puzzling behaviors observed in other rich search environments—are consistent with subjects adapting their search behavior in order to reduce memory load. The intuition for why these particular deviations lead to lower memory load is because they encourage just one alternative to be searched at a time, and make it easier to remember what has already been searched. By contrast, optimal sequential search, for example, will tend to “jump around” relatively more, between alternatives, in no particular spatial order, thus increasing the burden on memory. A possible mechanism for such adaptive behavior is that because humans have cognitive limitations, in the form of small finite working memory capacities, [20, 28–34], they wish to avoid increased memory load, as it will tend to lead to increased choice errors (due to memory failure). Therefore, in order to search in a way that reduces memory load (thus choice error), one is willing to deviate from the path of most valuable information (optimal sequential search).

This paper reports a novel experiment, designed to explore the effects of cognitive limitations on choice, in accordance with the predictions of the new model of search memory load introduced in [19]. In particular, it tests whether variation in solely the *order* in which information is acquired—which is irrelevant in standard models of choice, but alters the predicted working memory load in this model—predictably affects the error rate in subsequent choice.

An implication of the outcome of this test for search behavior, more generally, is that if increased memory load in search is found to increase choice error then this provides a structural explanation for why searchers adapt their behavior in the ways recently observed: in order to reduce memory load, and thus reduce the rate of choice error. On the other hand, if increased memory load in search is found to have no effect, or decrease choice error, then the case for memory limitations as an explanation of subjects’ systematic deviations from optimal sequential search will be weakened considerably.

The experimental design is as follows: in each task the subject chooses one of the available alternatives after all have been searched exhaustively. She is paid for a task only if she chooses the alternative with the highest revealed value. For simplicity, the value of each alternative is equal to the sum of its attribute values, which is a common way of approximating more naturalistic choice problems in the literature, while still allowing for an objectively correct choice [8, 10, 35–37]. The attribute values are random variables generated by a process that is explained clearly to subjects. The experimental manipulation of primary interest is the order in which subjects must search; the search order will be either within alternative intensive (called *AL*), because it searches each attribute in an alternative before moving to the next alternative, or within attribute intensive (called *AT*) because it searches the same attribute for all alternatives before proceeding to the next attribute. The intuition behind this manipulation is that AL search (almost always) requires less memory than AT search (as will be explained), and AL and AT are the two archetypal search orders found in the multiple attribute search literature (see [1, 7, 18]). The experimental manipulation of secondary interest is that matrices of attributes are of three different sizes—two alternatives by two attributes, 3×3, and 4×4. Each subject in the within subject design performs 60 search tasks—20 with each sized matrix, with half of the tasks for each matrix size AL and half AT. In order to control for a “western reading” effect, in half of the AL (AT) tasks alternatives are represented as rows, and in the other half as columns.

The model of working memory load (henceforth WML) introduced in [19] (Section 2) predicts that the maximum WML for AL is the same for all three matrix sizes (with a load of 3), while that of AT increases linearly from 3 to 5 to 7 as the matrix size increases from 2×2 to 3×3 to 4×4. The intuition for these differences is that AL search minimizes required WML by considering only one alternative at a time, while AT search must consider multiple alternatives at the same time (S2 Appendix shows how maximum WML’s are computed). Thus, it is predicted that in the experiment (1) in 2×2 matrices there will be no difference in subjects’ choice error rates between AL and AT search (because the maxiumum WML is the same for both), but for 3×3 and 4×4 matrices AL search will yield a lower rate of choice error than AT search (because the maxiumum WML is less), and (2) as the matrix size increases, the amount by which AT search yields larger choice error rates than AL search will also increase (because the difference in maxiumum WML increases).

The results are that, relative to AT search, AL yields no difference in the rate of correct choices in the 2×2 matrices, a larger rate of correct choices in the 3×3 matrices, and a much larger rate of correct choices in the 4×4 matrices. Thus, both predictions are supported by the data: when AL search requires less maximum WML it induces strictly lower rates of choice error, and as the difference in predicted maximum WML increases with matrix size, so does the disparity in choice error rates between the two search orders. These results provide strong evidence that the model of WML introduced in [19] is effective at tracking choice error rates, as the observed rate of choice error (for any matrix size) increases markedly with the predicted maximum WML in search. One implication of the results is that while the recently observed systematic deviations from the rational benchmark of optimal sequential search are costly in the sense that less valuable information is being searched, they are also beneficial in that the subsequent processing of acquired information is less prone to error. The discovery of evidence in support of this trade-off encourages the building and testing of behavioral models of optimal sequential search subject to memory limitations. A more general implication of these results is that, despite the focus in the information overload literature on the *amount* of information searched [2, 15, 38–43], the *order* alone that information is searched in is a fundamental determinant of cognitive load, thus choice behavior, independent of the amount of information searched.

This study also contributes to the cognitive load and cognitive skills literatures. Cognitive load experiments [44–62] have subjects perform a particular decision task while concurrently loaded with some other cognitively demanding task, such as remembering a seven digit number. The experiment reported here is similar, but induces cognitive load endogenously (rather than exogenously) within the decision task of primary interest. Experiments in the cognitive skills literature [46, 62–75] have subjects perform a decision task, and also perform separate tests of cognitive abilities; it is then checked if the test-based measure of a subject’s cognitive abilities is correlated with the subject’s performance in the decision task. The experiment reported here is similar, but implements a concurrent test of cognitive skills, and performance, within the decision task of interest. Thus, the effects of cognitive limitations on choice performance reported here are not correlational (as in the measures of cognitive ability), and not exogenous (as in cognitive load experiments), but direct, making this a novel contribution to these literatures.

Section 2 states the tested hypotheses formally. Section 3 provides the experimental design. Section 4 reports the results. Supporting Information S1 Appendix tests for effects related to western reading, task order, and the specific values searched. S2 Appendix demonstrates how to compute the WML of a search sequence. S3 Appendix provides the instructions used in the experiment. S4 Appendix gives an alternative formulation of the hypotheses which accounts for not only the maximum WML of a search sequence, but the aggregate WML as well.

## Hypotheses

A model of working memory load (henceforth WML) in search that imposes minimal structure on the memory process is introduced in [19]. The model takes any search sequence over a matrix of attributes, and predicts the WML for each step of the search sequence. Thus, the predicted maximum and aggregate WML’s of any search sequence are easily computed. Maximum WML tends to be examined exclusively in the cognitive psychology literature (see [31]) because it is the more cognitively binding of the two measures, so this approach will also be taken here. This model of WML predicts that the maximum WML of 2×2, 3×3, and 4×4 matrices of attributes, according to either AL or AT search, is as appears in Table 1. Recall that when each alternative is represented by a row of the rectangular matrix, AL search proceeds left-to-right, top-to-bottom, as in western reading. AT search, on the other hand, is AL search transposed, searching top-to-bottom, left-to-right.

**Table 1:** Maximum WML as a function of search order and matrix size.

Matrix Size	AL/AT	Max. WML
2×2	AL	3
2×2	AT	3
3×3	AL	3
3×3	AT	5
4×4	AL	3
4×4	AT	7

Table 1 shows that there is no separation in the predicted maximum WML between AL and AT search in the 2×2 matrix of attributes. However, in the 3×3 matrix there is a difference of two units of maximum WML, and in the 4×4 matrix there is a difference of four units of maximum WML. The intuition for why maximum WML remains constant for AL search, as the matrix size increases, is that it always evaluates just one alternative at a time. By contrast, as AT search evaluates all alternatives at a time, the WML increases with the number of alternatives.

Null and alternative hypotheses are formally stated here:

*H*1_0_: *AL*_2_ = *AT*_2_; *AL*_3_ = *AT*_3_; *AL*_4_ = *AT*_4_
*H*1_*A*_: *AL*_2_ = *AT*_2_; *AL*_3_ > *AT*_3_; *AL*_4_ > *AT*_4_,

where *O*_*m*_ is the relative frequency of correct choices of the search order *O* ∈ {*AL*, *AT*} for matrix size *m* ∈ {2, 3, 4}.

*H*2_0_: *AL*_2_−*AT*_2_ = *AL*_3_−*AT*_3_ = *AL*_4_−*AT*_4_
*H*2_*A*_: *AL*_2_−*AT*_2_ < *AL*_3_−*AT*_3_ < *AL*_4_−*AT*_4_.

Consistent with standard models of choice, the null hypotheses are based solely on the set of acquired information, indifferent to the order of acquisition. Thus, because the same information is acquired by both AL and AT for a particular matrix size, *H*1_0_ and *H*2_0_ follow immediately. By contrast, *H*1_*A*_ and *H*2_*A*_ take order into account, and directly follow from the levels of maximum WML predicted by the model in [19]: *H*1_*A*_ states that for any given matrix size, when AT requires the same maximum WML as AL (in the 2×2), then the two search orders yield identical relative frequencies of correct choices. However, when the maximum WML required of AT is higher than that required of AL for a particular matrix size (as in the 3×3 and 4×4), then the relative frequency of correct choices by AT will be lower. *H*2_*A*_ states that the amount by which AL outperforms AT, in terms of relative frequency of correct choices, increases monotonically with matrix size, as its WML advantage increases from 0 (in 2×2) to 2 (in 3×3) to 4 (in 4×4).

Hypotheses 1 and 2 allow pure tests of the effect of variable maximum WML in search on choice performance, without requiring that the choice process itself be modeled, as in both cases, only the order of search within the otherwise identical task is manipulated. This is true even as the matrix size increases. On the other hand, one may also be curious to study the effect of matrix size itself on choice error. Unfortunately, this is not as straightforward, as noise alone (independent of WML) will cause the error rate to increase with matrix size (because the fraction of alternatives that are incorrect increases). By contrast, noise cannot predict the effects conjectured in alternative hypotheses 1 or 2, though variable maximum WML does. Because the purpose of this study is to test the effect of predicted maximum WML (alone) on choice error, such hypotheses regarding a pure matrix size effect are left for future work (see the results section for a bit more on this).

## Methods

The experiment was conducted in the LaTEx laboratory of the University of Alicante, Spain. 58 subjects were chosen at random from a pool of 878 students who had recently registered to participate in decision making experiments. Each of the subjects also voluntarily signed up for participation in a particular session. At the beginning of each session, subjects were given written instructions, which were also read aloud by an experimenter, in which they were fully informed about the content of the experiment (an arithmetic task for which they could only earn positive amounts of money), reminded that no deception of any kind can be used in economics experiments, and notified that they would be free to abandon the experiment and leave at any point (though none chose to do this). The author only interacted with subjects in reading the instructions, answering any questions (individually) immediately following the instructions, and paying subjects after the experiment. Further, data was anonymized before being obtained by the author (in accordance with Spanish Law 15/1999 on personal data protection).

The content and procedure of the experiment was checked and approved by the University of Alicante’s Dean of Business and Economics, who was in no other way involved in the study. At the time the experiment was conducted there was no official IRB committee established at the school of Economics.

The experimental design was within subject; each subject responded to 60 search tasks, using the Mouselab experimental interface. Each search task contained either a 2×2, 3×3, or 4×4 matrix of attribute values. In some tasks each alternative was represented by a row of attributes, and in others by a column. The value of each alternative was determined by the sum of its attributes, which was initially unknown to the subject, but fully revealed over the process of search. Attribute values were displayed to the subject one at a time, for as long as the subject liked, but in an order determined by the experimenter. Rushing was discouraged by requiring subjects to stay in the laboratory for at least 40 minutes once the search tasks began, which was plenty of time to complete all of the tasks at a casual pace.

Once the subject had searched each attribute of the matrix once (with no repeat searches allowed) she was asked to choose an alternative. Subjects were instructed that in each task one of the alternatives had the unique largest value. Also, at the end of the experiment, four of the 60 tasks would be chosen at random, and four euros would be paid for each of those tasks in which the highest valued alternative had been chosen; otherwise no money would be paid for that task. This variable reward was added to a show up fee of five euros.

While subjects initially did not know the values of attributes, the random process which generated the values was explained to them. The main objective of the random process designed by the experimenter was to make it difficult for the subject to choose the highest valued alternative at a rate better than chance, without assimilating each and every searched attribute value. This was emphasized to subjects in the instructions. Because the predicted memory load changes according to search order, and search order is manipulated directly by the experimenter, holding all else constant (matrix size, attribute values, etc.), the design allows for a pure test of the effects of predicted memory load on choice performance.

The random process used to generate the matrices of attributes is as follows. First each attribute value in a 2×2, 3×3, or 4×4 matrix is independently drawn from a uniform distribution over the integer values from -10 to 10. In order for such a matrix to then be used in the experiment, it must survive three filters. The first filter requires that the first attribute of each alternative, and the final sum value of each alternative, have a unique value. First attributes are required to be unique, because otherwise the subject could “chunk” [30] the memory of the non-unique value for multiple alternatives, thus effectively reducing experienced WML beneath the value predicted by the model. The final sum values of each alternative are unique in order to provide the most precise target for correct choice. The second filter requires that differences in the running alternative sums between any two alternatives never exceed 5 after the same subset of attributes have been searched for each of the alternatives. Requiring the alternatives to be in a “close race” makes it unappealing for the searcher to stop attending to a particular alternative because it is “too far behind,” which could otherwise serve to reduce experienced WML beneath the amount predicted by the model. The third and final filter gives subjects further incentive to attend to all information by reinforcing that the current best alternative need not remain the best as more information is acquired. In particular, after the same strict subset of attributes have been searched for each of the alternatives, it is equally likely that as additional attributes are searched for all alternatives, the current highest valued alternative will change positions any number of times (between zero and the number of attributes then remaining for each alternative). For example, if a task has three alternatives, and after searching the first attribute for each of the alternatives, the current highest valued alternative is the first, then as the second and third attributes are searched for all three alternatives it is equally likely that the position of the highest valued alternative will switch once, twice, or not at all. It was emphasized to subjects that the purpose of the filters was to make it hard for them to do well at the task without attending to all of the attributes in the matrix, and that this was all they really needed to understand. If they then wanted to pay attention to the specifics of the data generating process they were able to, as the instructions were read aloud to all. For more details see the instructions in S3 Appendix.

**Fig 1:**
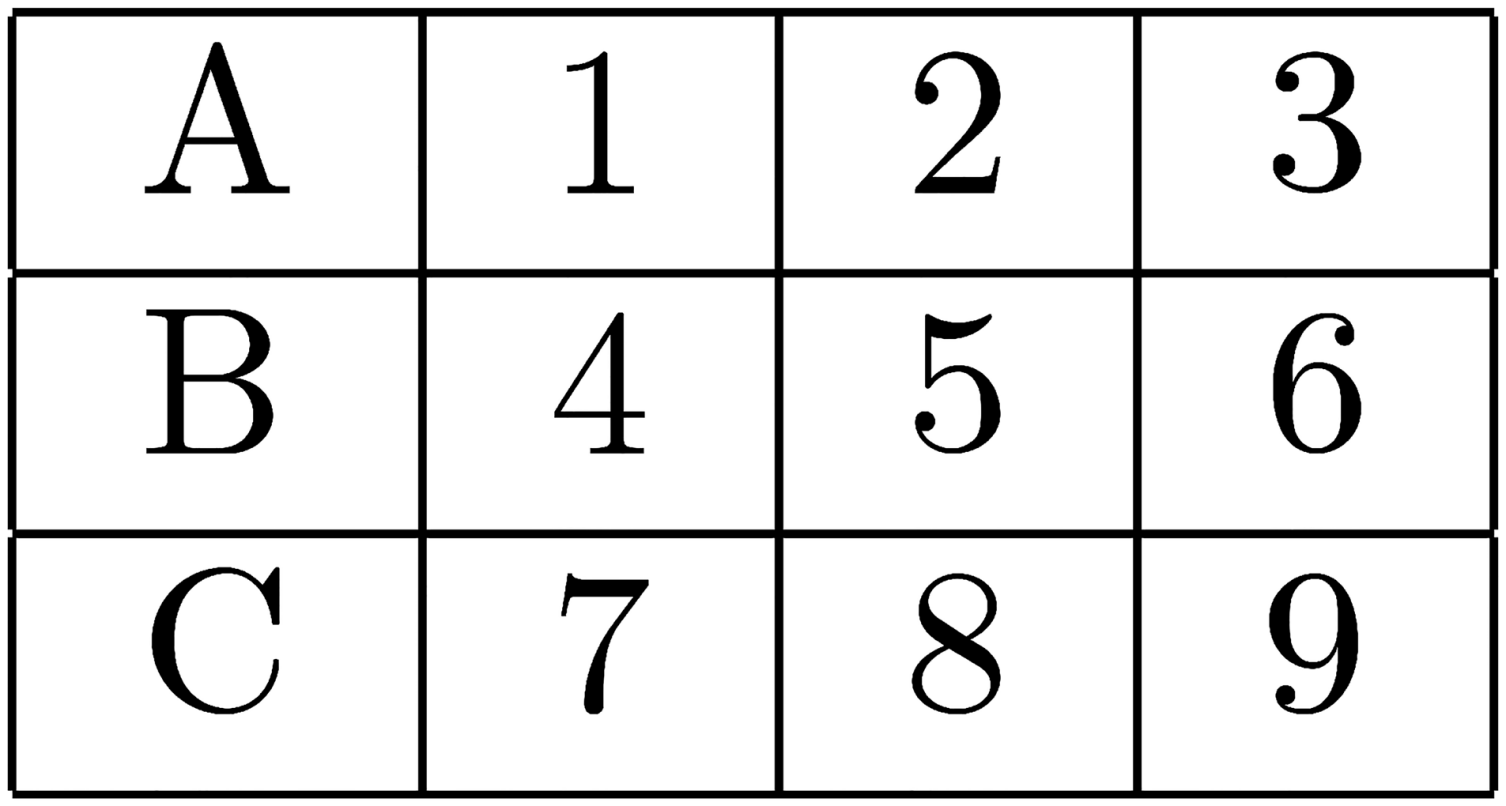
AL search order when alternatives are configured as rows.

**Fig 2:**
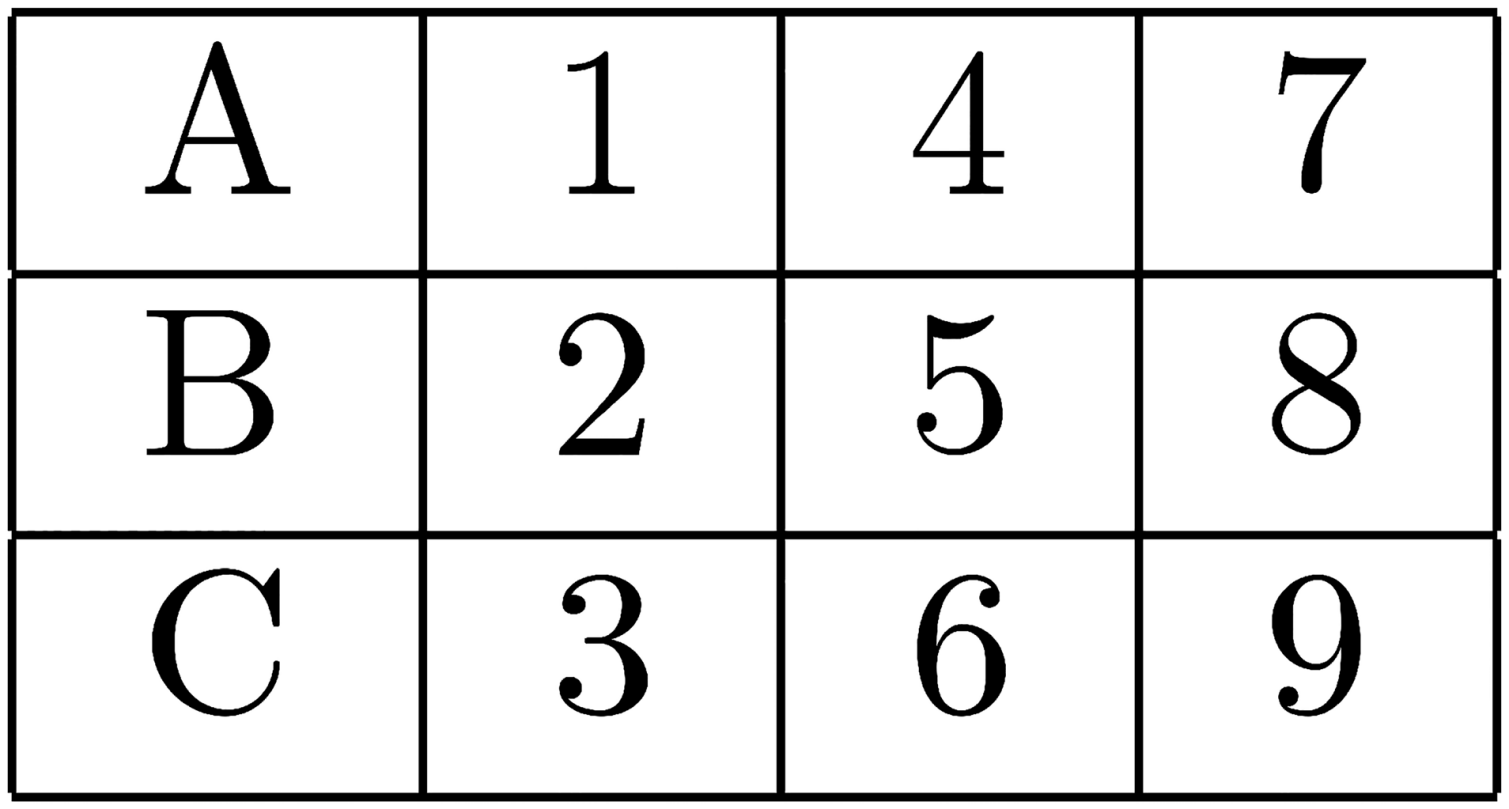
AT search order when alternatives are configured as rows.

**Fig 3:**
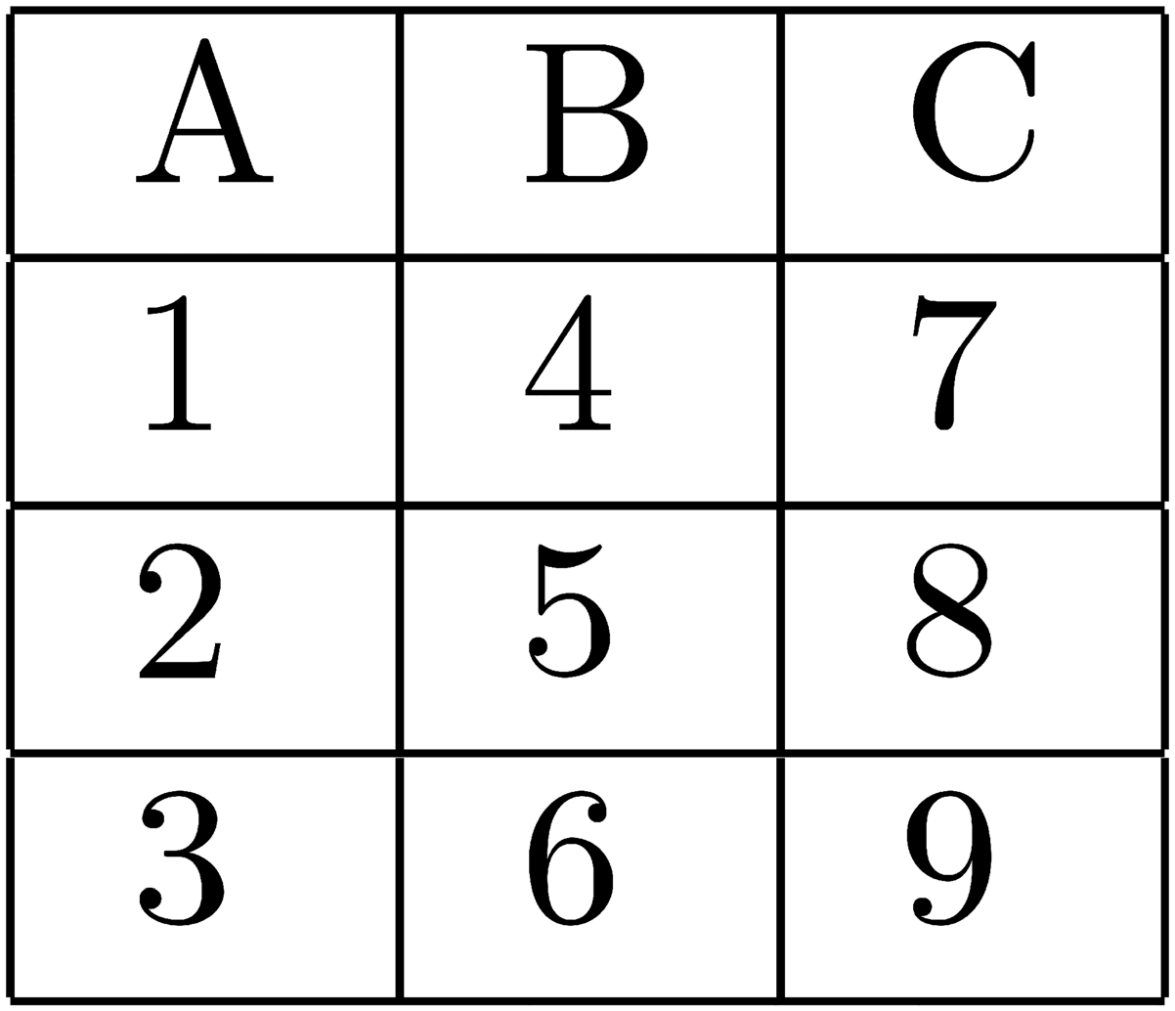
AL search order when alternatives are configured as columns.

**Fig 4:**
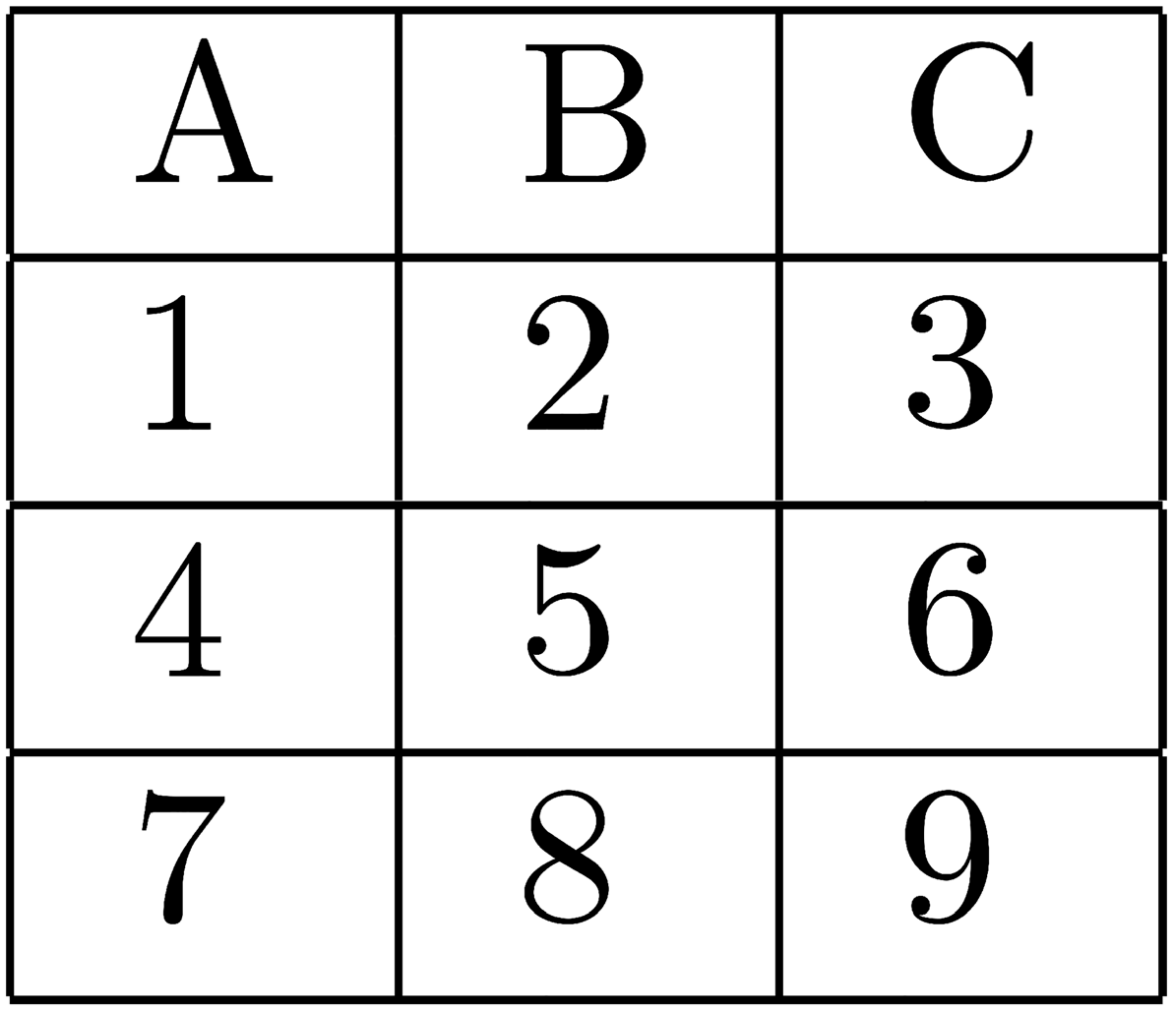
AT search order when alternatives are configured as columns.

There were two primary experimental manipulations. The first was that the imposed search order could be either AL or AT in any given task (see Figs 1–4 below). The second was that the size of the matrix of attributes varied from two alternatives by two attributes, to 3×3, to 4×4. The purpose of these two manipulations was to allow a clear test of the effects on choice error of variable predicted WML, as a function of search order. The alternative hypotheses presented and explained in the previous section reflect the predictions made by the model of WML in [19], as explained in more detail in S2 Appendix.

Of the 60 search tasks that each subject responded to, 20 contained 2×2 matrices of attributes, 20 contained 3×3’s, and 20 contained 4×4’s. For each matrix size, 10 tasks required AL search and 10 required AT search. 5 of the 10 tasks of each type had alternatives represented as rows in the matrix of attributes, and 5 had the alternatives represented as columns. See the AL and AT search orders for 3×3 matrices with alternatives as rows (columns) in Figs 1(3) and 2(4), respectively. Search orders are similar for 2×2 and 4×4 matrices.

The 60 different tasks were randomized in one order for half of the subjects, and in another order for the other half of subjects. This allowed for a test of a task order effect. Further, as half of the AL (AT) tasks for each subject had alternatives represented as rows, and the other half as columns, a possible relative facility of one search pattern over the other (western reading vs. transposed western reading) was controlled for. Finally, a possible effect due to the particular attribute values in each task was controlled for by implementing a counter-balancing scheme. Half of the subjects would search any particular matrix of attributes in the AL order while the other half would search the same matrix of attributes in the AT order.

## Results

Both of the novel predictions based on the model of WML in [19] are supported by the data. In particular, all things equal, as the predicted WML of a search order increases, the relative frequency of correct choices falls. Also, as the predicted differences in AL and AT’s WML’s increase with matrix size, the differences in the relative frequencies of correct choices also increase. Effect sizes are substantial.

**Fig 5:**
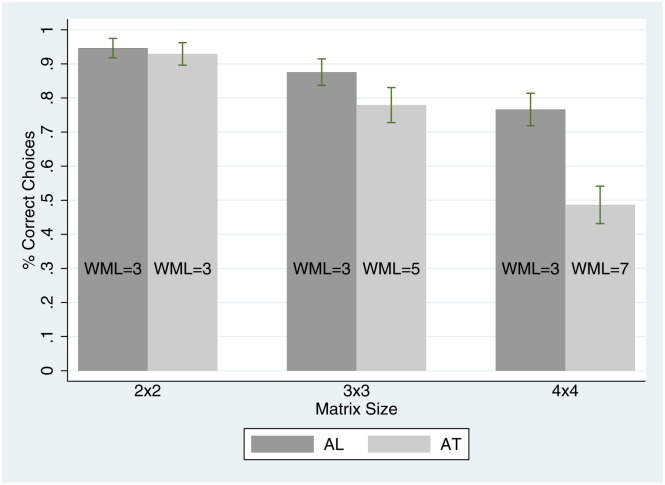
Relative frequency of correct choices as search order and matrix size vary.

Fig 5 reports relative frequency of correct choices, averaged across subjects (with standard errors), as predicted WML varies with search order and matrix size. Both hypotheses stated in the second section are tested using a repeated measures ANOVA, with two repeated factors: search order and matrix size. *H*1 consists of three tests: one test of the simple effect of search order for each matrix size. As is clear from the hypotheses section, for matrix size 2 the test is two-sided, whereas for matrix sizes 3 and 4 the tests are one-sided. In order to address the multiple comparison issue the Bonferroni correction is used, as described below. In the 2×2 matrices of attributes in which AL and AT searchers experience the same predicted amount of maximum WML, AL searchers are shown to make the correct choice in 94.66% of tasks while AT searchers make the correct choice in 92.93% of tasks; this difference is not statistically significant (0.0172 [0.0214], p = .423), where the numbers enclosed in parentheses are (average difference [standard deviation], and p-value]. Meanwhile, in the 3×3 matrix, where AL searchers are predicted to experience less maximum WML than AT searchers (3 vs. 5, respectively) AL searchers make the correct choice in 87.59% of tasks, while AT searchers make the correct choice in 77.93% of tasks; this difference is statistically significant (0.0966 [0.0214], p < .001). Finally, in the 4×4 matrix, where AL searchers are predicted to experience much less maximum WML than AT searchers (3 vs. 7, respectively) AL searchers make the correct choice in 76.63% of tasks, while AT searchers make the correct choice in 48.66% of tasks; this difference is statistically significant as well (0.2797 [0.0214], p < .001).

*H*2 tests the interaction between search order and matrix size, in two pairwise comparisons, by matrix size. As clear from the hypotheses section, both pairwise tests are one-sided. As seen above, in the 2×2 matrix the difference in relative frequency of correct choice between AL and AT search orders is 1.72% (the difference in predicted maximum WML is 0), while the difference goes up to 9.66% in the 3×3 matrix (where the difference in predicted maximum WML is 2), yielding a significant difference in differences (0.0793 [0.0303], p = .005). Similarly while the difference in the 3×3 matrix is 9.66%, the difference in the 4×4 is 27.97% (where the difference in predicted maximum WML is 4), yielding another significant difference in differences (0.1831 [0.0303], p < .001). Between the two hypotheses five total pair-wise tests are conducted—three for the first hypothesis, and two for the second, thus with the Bonferroni correction the significance threshold for all pairwise tests becomes 0.01. Given the p-values reported one can see immediately that with the correction all one-sided test statistics remain highly significant, and the two-sided test statistic remains insignificant. Finally, two of the 60 tasks were removed from the data, due to a minor data entry error by the experimenter. Namely, the sign of an attribute value was switched in each of the two tasks. Relative frequencies of correct choice with or without these tasks removed are nearly identical, and results of hypothesis tests are identical.

Thus, both predictions are supported by the data: as the predicted maximum WML of a search sequence increases, all else equal, so too does the relative frequency of choice error. Also, as the differences in the predicted maximum WML’s between two search orders of the same matrix size increase, so too do the differences in the relative frequencies of correct choices.

The experimental design also allowed for the testing of several “second order” effects: task order effects, western reading effects, and effects of the particular values of attribute matrices. No evidence of any such effect is discovered. See S1 Appendix for details.

At the end of the hypotheses section it is explained that a test of the effect of matrix size on choice error is omitted, as the role of maximum WML in such a test would have to somehow be disentangled from a pure noise effect. By contrast, tests of hypotheses 1 and 2 are not vulnerable to this issue. Having said this, one can observe that the effect of matrix size on error rate is positive (and significant). Though noise alone can explain this effect, a rough proxy measure that one might consider including for the practical purpose of predicting choice error rates across matrix sizes is aggregate WML, which for AL search, for example, does reflect matrix size in its measure (unlike maximum WML). One can see both of these measures side-by-side, for each search order and matrix size, in Table B of S2 Appendix. It is at least intriguing that while the correlation between maximum WML and observed choice error rates, across the six within subject conditions, is.904, the correlation between aggregate WML and choice error rates is.999.

## Conclusion

While standard models of choice disregard the *order* in which information is acquired, the model of working memory load (WML) in search introduced in [19] predicts that order is fundamental to experienced memory load. Because working memory capacity has long been recognized by psychologists (and more recently by neuro-scientists) as a fundamental bottleneck in decision-making, this paper tests, in an experiment, the effects of search order, thus predicted WML, on the rate of choice error. The model of WML is found to track choice error rates remarkably well; the higher the predicted WML of a given search sequence, despite the same information being searched, the higher the observed rate of choice error. These results provide a mechanism for recently observed systematic deviations from optimal sequential search: that subjects search too deeply within alternatives (at the expense of searching other alternatives), and switch too adjacently between alternatives, systematically reduces experienced levels of WML. When the results reported in this paper are combined with these observed fact patterns, as well as others from a variety of rich search contexts (see [19]), what is suggested is an adaptive form of rationality in which boundedly rational searchers deviate from optimal sequential search (which assumes unlimited memory) in order to reduce experienced WML, and thus choice error. Consequently, these results encourage the building and testing of behavioral models of multiple attribute search, in which optimal sequential search is subject to memory limitations.

In order to study the effect of cognitive load on decision making, experiments in the cognitive load literature impose a concurrent (exogenous) load task on the subject, which is separate from the task of interest. Experiments in the cognitive ability literature measure performance in one task and test how this correlates to decision making behavior in another. By contrast, the experimental design employed in this study manipulates cognitive load directly (endogenously), within the task of interest. Thus, because the discovered effects of cognitive limitations (memory) on choice behavior are found to be substantial, and are neither exogenous, nor correlational, they constitute a contribution to both literatures.

The information overload literature has traditionally focused on the quantity of information presented to a decision-maker. The results reported here indicate that even when the quantity of information is held constant, the order that information is attended to in is a critical determinant of cognitive load, and thus choice behavior. Because experimenters typically have the ability to control or monitor the order in which information is provided to subjects, and because internet search data is becoming increasingly available, the predictive success of the model of WML tested in this paper suggests that it should not only be used in new behavioral search models, but also as a diagnostic tool that can be applied in both the lab and the field, in order to help understand the causes of consumer welfare, confusion, overload, and choice error. To provide one example, it offers an exclusion criterion of the type called for in the extended choice literature [76, 77]: if information is searched in a high (low) WML order then observed choices can be expected to be more (less) error-proned, thus deemed less (more) representative of underlying preferences (e.g. if Jon chooses alternative A following a high WML search order, and B following a low WML search order, then the researcher can weigh choice A relatively less).

One potential concern regarding the generalizability of the current results to a broader, and perhaps more natural, set of choice tasks, is that the experimental choice task employed is quite abstract, simplifying the evaluation of each alternative’s attributes by representing them as numerical values, and simplifying the integration of attribute values by making the value of each alternative equal to the sum of its attributes. Nevertheless, this approach is fairly standard among economists (see, e.g. [8, 37]), as it is considered a reasonable approximation of more naturalistic choice tasks, but with the relative benefit of allowing an objective measure of correct choice. Further, because the intuition underlying the model of WML is simply that evaluating multiple alternatives at the same time requires a greater memory load than evaluating one at a time, it is reasonable to expect the effect of memory load on choice error rates observed in the present task to hold more generally. Indeed, a recent eye-tracking study in a natural choice environment, in which computers are evaluated over multiple attributes [1], reports that subjects find within alternative intensive (AL) search to be easier than within attribute intensive (AT) search, both in terms of assimilating information and making decisions, and are more confident in their choices when they start by searching in this way.

## Supporting Information

S1 Appendix Tests for effects of western reading, task order, and the specific values searched. (PDF)

S2 Appendix How to compute the WML of a search sequence. (PDF)

S3 Appendix Instructions used for the experiment. (PDF)

S4 Appendix Alternative formulation of the hypotheses based on both maximum and aggregate working memory load. (PDF)

S5 Appendix Brief review of multi-attribute search theory literature. (PDF)

S1 Other Data. (XLS)

S2 Other Program to analyze data. (TXT)

S3 Other README file describing data. (TXT)
