# Supplementary material for: Search, Memory, and Choice Error: An Experiment
Source: PLoS One. 2015 Jun 29;10(6):e0126508. doi: 10.1371/journal.pone.0126508 (PMC4487248; doi:10.1371/journal.pone.0126508)
Supplement: S1 Appendix — (PDF) [file pone.0126508.s001.pdf]

Appendix S1: Secondary Tests

The experimental design also allowed for the testing of several "second order" effects: task order effects, western reading effects, and effects of the particular values of attribute matrices.

The tasks were randomized in one order for half of the subjects, and in another order for the other half. There is no order effect in the relative frequency of correct choices: 79.97% vs. 80.68%, as the difference is statistically insignificant (0.71%, p = .858).[1]

A test for a "western reading effect" was made possible by having alternatives represented by rows of the matrix of attributes for half of each subject's tasks, and columns in the other half of tasks. Presumably, subjects may perform differently when they are searching the matrix of attributes in the same pattern that they read a book in, as opposed to this search order transposed. Nevertheless, there is no difference in performance: 80.46% vs 80.15% (0.31%, p = .901).[2]

While the same 60 tasks were used for all subjects, half of the subjects searched any given task in the AL order while the other half searched the task in the AT order. In this way the particular values drawn in attribute matrices could be controlled for while the search order was manipulated. A check of performance, comparing the two halves of subjects shows no statistically significant difference in the relative frequencies of correct choices: 80.00% vs. 80.67% (0.67%, p = .834).[3]

[1] As compared samples are independent, a Mann-Whitney test is employed. An unpaired t-test yields similar results.

[2] As compared samples are not independent, a Wilcoxon signed-rank test is employed. A paired t-test yields similar results.

[3] As compared samples are independent, a Mann-Whitney test is employed. An unpaired t-test yields similar results.
