# Supplementary material for: Search, Memory, and Choice Error: An Experiment
Source: PLoS One. 2015 Jun 29;10(6):e0126508. doi: 10.1371/journal.pone.0126508 (PMC4487248; doi:10.1371/journal.pone.0126508)
Supplement: S2 Appendix — (PDF) [file pone.0126508.s002.pdf]

Appendix S2: How to Compute WML

The predicted *total WML* required in any search problem is equal to the sum of all *value* and *location* representations logically required to compute the value of, and locate, the highest valued alternative.

First examples will be used to show how to compute the predicted WML of values, then the predicted WML of locations, then the total predicted WML. For a more detailed description, please see [1].

In each example the predicted WML required for each step of the search sequence is presented in the corresponding position of the information matrix. The type of search being modeled is uncontingent rather than contingent, because the order of search is determined by the experimenter rather than by attribute realizations.

A few bits of notation necessary to read the computations below are

t: a counter of the step in a given search sequence; $t = 1$ corresponds to the first cell searched, $t = 2$ to the second, and so on. A search sequence visits each cell exactly once.

M: the number of alternatives (rows) in the information matrix.

N: the number of columns of attributes.

v_{mn}: value in the matrix corresponding to row m and column n, where $m \in \{1, 2, 3, \ldots, M\}$ and $n \in \{1, 2, 3, \ldots, N\}$.

$WML^{\cdot V}$: WML of values,

$WML^{\cdot L}$: WML of locations,

$WML^{\cdot T}$: $WML^{\cdot V} + WML^{\cdot L}$ (total WML),

$WML^{U\cdot}$: WML of uncontingent search

Uncontingent WML of Values

Figure 1. WML of Values: AL

0	1	1	1
1	2	2	2
1	2	2	2
1	2	2	2

$$\max_{\{t\}}\{WML^{UV}(t|AL)\} = 2 = 2$$

$$\sum_{t=1}^{MN} WML^{UV}(t|AL) = 2MN - N - M = 24$$

Figure 2. WML of Values: AT

0	4	4	4
1	4	4	4
2	4	4	3
3	4	4	2

$$\max_{\{t\}}\{WML^{UV}(t|AT)\} = M = 4$$

$$\sum_{t=1}^{MN} WML^{UV}(t|AT) = M^2(N-1)+(M-1) = 51$$

In each of the examples given in Figures 1 and 2 there are four alternatives and four attributes. Call the value in the top left corner v_{11}, that directly to the right of it v_{12}, that directly beneath it v_{21}, and so on.

For the AL sequence depicted in Figure 1 search starts with v_{11}. Initially, zero WML^{UV} is required because the value v_{11} is visible. However, once search proceeds to v_{12}, now the value of v_{11} must be remembered because it is no longer visible.[1] As v_{12} is being observed its value is added to the remembered value of v_{11}. Then search proceeds to v_{13}, but now with the single summed value (v_{11} +v_{12}) remembered; this process iterates until v_{14} is added to the running sum (v_{11} +v_{12} +v_{13}). When v_{21} is next searched the single summed value (v_{11} +v_{12} +v_{13} +v_{14}) must be remembered. Search then transitions to v_{22}, and

[1] In many information board designs only one attribute is visible at a time. In an environment where all attributes are visible at once this model corresponds to a human being who can attend to at most one attribute in any given instant, which is the simplest and most natural way to model deliberate attention.

now the value of v_{21} must be remembered in addition to the sum value of the top alternative, and so on. Once two alternatives have been exhaustively searched the sum values are compared and the low value is dropped from memory. Crucially, regardless of how many alternatives have been exhaustively searched, only one of these alternative's values need be recalled—the highest.

The maximum WML^{UV} for any step of the AL search sequence is thus two, for arbitrarily large numbers of alternatives and attribute columns. The aggregate WML^{UV}, in general, is $2MN - N - M$, which in the four by four is 24.

For the particular AT sequence depicted in Figure 2, search also starts with v_{11}, but then proceeds to v_{21}. Now that v_{11} is no longer observed, it must be remembered. Search then transitions to v_{31}, and now both v_{11} and v_{21} must be remembered. When the search sequence arrives to v_{12} four values must be remembered: v_{11} ,v_{21} ,v_{31} ,and v_{41}. This WML^{UV} of four persists until v_{34} is reached, at which point two alternatives have now been searched exhaustively, so the lower of these two alternative values can be discarded, which means a WML^{UV} of three rather than four, and for the search of v_{44}, two rather than three.

The key intuition for the differences in WML^{UV} between the AL and AT sequences is that WML^{UV} is equal to the number of alternatives that have been partially, but not exhaustively searched.

Uncontingent WML of Locations

Figure 3. WML of Locations: AL

0	0	0	0
0	1	1	1
1	1	1	1
1	1	1	1

$$\max_{\{t\}}\{WML^{UL}(t|AL)\} = 1 = 1$$

$$\sum_{t=1}^{MN} WML^{UL}(t|AL) = (M-1)N - 1 = 11$$

Figure 4. WML of Locations: AT

0	3	3	3
0	3	3	3
1	3	3	2
2	3	3	1

$$\max_{\{t\}}\{WML^{UL}(t|AT)\} = M - 1 = 3$$

$$\sum_{t=1}^{MN} WML^{UL}(t|AT) = (N-1)(M-1)M = 36$$

For AL (Fig. 3), the searcher need not remember any locations until there are two distinct alternative values to be recalled simultaneously.[2] This occurs for the first time as she searches the sixth attribute in the sequence. Remembering which alternative corresponds to one of the two values is sufficient for her to identify which alternative the other value belongs to.[3]

For AT (Fig. 4), The WML of locations (WML^{UL}) is similar to its WML^{UV} (see Fig. 2), but consistently one unit lower due to a searcher's ability to always logically identify the values associated with each of m alternatives by remembering the spatial locations of only $m - 1$ of those values.

The basic intuition regarding the respective WML^{UL}'s of AL and AT search sequences is the same as it was for WML^{UV}; AL has the lowest possible number of alternatives open for each step of the search sequence, while AT has the highest.

Figure 5. Total WML ($WML^{UV} + WML^{UL}$): AL

0	1	1	1
1	3	3	3
2	3	3	3
2	3	3	3

$$\max_{\{t\}}\{WML^{UT}(t|AL)\} = 3 = 3$$

$$\sum_{t=1}^{MN} WML^{UT}(t|AL) = 3MN-2N-M-1 = 35$$

Figure 6. Total WML ($WML^{UV} + WML^{UL}$): AT

0	7	7	7
1	7	7	7
3	7	7	5
5	7	7	3

$$\max_{\{t\}}\{WML^{UT}(t|AT)\} = 2M - 1 = 7$$

$$\sum_{t=1}^{MN} WML^{UT}(t|AT) = 2M^2(N-1)+M(2-N) = 87$$

Uncontingent Total WML

Figures 5 and 6 show total WML ($WML^{UT} = WML^{UV} + WML^{UL}$) for the uncontingent search sequences AL and AT, respectively, combining the WML's required for both values and locations. By plugging in the appropriate values of M (number of alternatives) and N (number of attributes) the reader can reproduce maximum (and aggregate) total WML for AL and AT search sequences, for matrices of any size.

References

1. Sanjurjo A (2014b) The role of memory load in search and choice. Working paper.

[2] The order of an uncontingent search sequence follows a search rule, thus by knowing where she is currently searching, the searcher knows where she has already searched.

[3] Throughout, it is assumed that searchers only store necessary information (optimal discard).
