# Supplementary material for: Search, Memory, and Choice Error: An Experiment
Source: PLoS One. 2015 Jun 29;10(6):e0126508. doi: 10.1371/journal.pone.0126508 (PMC4487248; doi:10.1371/journal.pone.0126508)
Supplement: S3 Appendix — (PDF) [file pone.0126508.s003.pdf]

Appendix S3: Instructions

Overview Welcome!
Today you will be asked to make a series of choices, for which you will be rewarded more or less money depending on the choices you make. I expect the average participant to earn around 16 euros but it is possible to earn as much as 21 or as little as 5, depending on the choices you make. You should expect to be here for around one hour. You will be paid as you leave.[1]

You will make 60 choices in all; for each choice you will choose one alternative from a set of either two, three, or four alternatives.

I will now give more details about the choices you will be making and how you will be paid, then you will see a demo with three examples of exactly what your choice tasks will look like, then you will start the series of 60 paid choice tasks. After you finish the paid choice tasks you will be asked to fill out a questionnaire. After the questionnaire you will be paid. As we go through the instructions please know that there is a strict culture of honesty in economics experiments, so you will not be mislead or deceived in any way. It is also very important that you do not communicate with anyone until after you have finished the experiment and left the room. Also you will not be able to use paper and pencil, or anything other than what you are instructed to use on the computer display. If any of these conditions are violated we will unfortunately have to ask you to leave and you will not be paid.

More about the tasks For each task you will need to choose from a set of either two, three, or four alternatives. Each alternative contains multiple characteristics, and the value of each alternative is simply the sum of its own characteristics. The way to earn the most money is to choose the alternative with the highest value in each task. When there are two alternatives to choose from each alternative consists of two characteristics, when there are three alternatives each consists of three characteristics, and when there are four alternatives each alternative consists of four characteristics. In any choice task, the alternatives can either be represented as rows or columns, for the duration of the task. An example of the two different ways alternatives can be represented is shown directly below:

alternative a	alternative b	alternative c

alternative a		
alternative b		
alternative c		

A very important feature of the tasks is that rather than see all characteristic values at the same time, instead **you will see only one characteristic value at a time.** Consider a two alternative choice task in which the alternatives are represented as rows and characteristic values are shown according to Order 1. The four pages you would see in sequence would look like this:

[1]The instructions provided here are an English translation (from Spanish) of the instructions given to the students that participated in the experiment. The author would like to thank Maria José Aragón for doing the bulk of the translation.

Page 1:

alternative a	0	
alternative b		

Page 2:

alternative a		5
alternative b		

Page 3:

alternative a		
alternative b	-2	

Page 4:

alternative a		
alternative b		6

You will observe each page once and only once, and you will control when you go to the next page by clicking on a button labeled “next page” on the bottom of the page.

For expositional purposes I now show all characteristic values together (though you will never see this in the experiment) along with calculated alternative values.

alternative a	0	5
alternative b	-2	6

Value of alternative a $= 0 + 5 = 5$
Value of alternative b $= -2 + 6 = 4$

For each of the two, three, and four alternative tasks there are two distinct orders that the characteristic values can be shown to you. You will be told throughout each task which order the characteristic values will be shown to you in (so you do not have to remember the orders). The two orders that characteristic values can be shown to you in the two alternative task, for example, are

Order 1:

alternative a	1-first	2-second
alternative b	3-third	4-fourth

alternative a	alternative b
1-first	2-second
3-third	4-fourth

Or
Order 2:

alternative a	1-first	3-third
alternative b	2-second	4-fourth

alternative a	alternative b
1-first	3-third
2-second	4-fourth

The two orders in the three and four alternative tasks are similar.

Notice that there are three things that can change from one choice task to the next: 1. the number of alternatives (thus characteristics also) 2. whether the alternatives are represented as rows or columns 3. which of the two orders the characteristic values will be shown in

You will choose in 20 two alternative choice tasks, 20 three alternative choice tasks, and 20 four alternative choice tasks. For each number of alternatives 10 choice tasks will have alternatives represented as rows, and 10 as columns. Further, 5 of the 10 choice tasks in which alternatives are columns will have order 1 and five will have order 2; the same is true of choice tasks in which alternatives are represented as columns. The order of these choice tasks was chosen randomly before the beginning of the experiment.

Characteristic Values The characteristic values for each choice task can be at lowest -10 and at highest 10. They were chosen using a random method designed to make it difficult to guess which alternative has the highest value without summing all of the observed characteristic values for each alternative.

In case you want more details on how the characteristic values were generated (optional):

– First, all characteristic values (integers) between -10 and 10 are drawn with equal probability, independently (so the drawing of any particular characteristic value in no way affects draws of other characteristic values).

Then, in order to be selected as the characteristic values for one of your choice tasks the values also had to comply with the following three rules:

– The maximum difference between any two temporary alternative value sums is five.

– It is equally likely that the temporary highest alternative value sum changes any number of times after the temporary alternative value sum that consists of one characteristic only. For example, in a four alternative task, if alternative A is the temporary highest alternative value sum after seeing only the first characteristic value for each alternative, then as temporary sums including two, three, and four characteristic values are compared, it is equally likely that the highest temporary alternative value sum changes zero, one, two, or three times.

– Initial and final alternative value sums are never equal across alternatives.

If this seems confusing do not worry. What is important is that you understand that it is difficult to ”guess” the highest alternative value by considering only some of the characteristic values. The only way to ensure that you select the highest value alternative and report its correct value is by using all characteristic values.

Payment You will be paid 5 euros for simply showing up and following the instructions of the experiment. In addition, after you have finished we will check the answers for 4 tasks that were chosen at random before the start of the experiment (we will show you the numbers from inside of a sealed envelope that we will open). For each of these 4 tasks you will be paid 4 euros for a correct answer. Thus, the most money you can earn is 21 euros, and the least is 5.

Please keep in mind that you will need to remain in your seat, at your computer for at least 40 minutes once your choice tasks begin, in order to be eligible for payment. Thus, there is no reason to rush through the experiment. Once at least 40 minutes have passed (we will indicate this clearly on the chalk board) you may raise your hand to indicate that you have finished the decision tasks and the questionnaire. Please wait silently while we calculate your payoff; this may take a few minutes. Once we have calculated your payoff we will call you one by one and lead you outside of the room where we will pay you according to your performance in the 4 tasks chosen at random; also, we will give you a sheet of paper indicating which of these tasks you answered correctly and which you answered incorrectly. If you would like to verify incorrect answers, or the numbers selected at random to determine the 4 paid tasks, you need only ask us to see the official data when we are giving you your payment.

Summary Remember, throughout each choice task you will know how many alternatives you are choosing between, whether the alternatives are represented by alternatives or columns, and what order the characteristic values will be shown to you in. The more choices in which you choose the highest value alternative, the higher you can expect your earnings to be. As the experiment will require a good deal of attention, please make an effort not to distract other students during the duration of the experiment.

Examples We will now go through three examples in order to see more precisely what the choice tasks you will be seeing look like. Unlike when your choice tasks begin, for these examples we will show the

calculated value of each alternative, and thus which alternative value is higher.

After finishing the demo you will have an opportunity to ask questions before starting the paid portion of the experiment. It is important that you ask any questions that you have then as you will not be able to ask questions once the paid portion of the experiment begins.

We will now start the demo.
