# Supplementary material for: Search, Memory, and Choice Error: An Experiment
Source: PLoS One. 2015 Jun 29;10(6):e0126508. doi: 10.1371/journal.pone.0126508 (PMC4487248; doi:10.1371/journal.pone.0126508)
Supplement: S4 Appendix — (PDF) [file pone.0126508.s004.pdf]

Appendix S4: Hypotheses with Aggregate WML

While the working memory load literature in cognitive psychology is sharply focused on the maximum WML incurred during the decision making process [1], the model of WML in search introduced by [2] allows also for computing the estimated aggregate WML. Table 2 shows both the maximum and aggregate WML's for the AL and AT search orders in matrices of attributes of size two by two, three by three, and four by four.

Table 1. Maximum and aggregate WML as a function of search order and matrix size

Matrix Size	AL/AT	Max. WML	Aggregate WML
2x2	AL	3	5
2x2	AT	3	7
3x3	AL	3	17
3x3	AT	5	32
4x4	AL	3	35
4x4	AT	7	87

Aggregate WML may be useful as a second order measure of memory load, given that it measures sustained working memory load. If it were believed that aggregate WML might have even a weak effect on predicting the relative frequency of correct choices, then a revised pair of hypotheses from the hypotheses section could look like the following:

$H1_0 : AL_2 = AT_2;\ AL_3 = AT_3;\ AL_4 = AT_4$
$H1_A : AL_2 \geq AT_2;\ AL_3 > AT_3;\ AL_4 > AT_4,$

where O_m is the relative frequency of correct choice of the search order $O \in \{AL, AT\}$ for matrix size $m \in \{2, 3, 4\}$

$H2_0 : AL_2 - AT_2 = \ AL_3 - AT_3 = \ AL_4 - AT_4$
$H2_A : AL_2 - AT_2 < \ AL_3 - AT_3 < \ AL_4 - AT_4,$

Thus the only change relative to the hypotheses presented in the second section, which only depend on maximum WML, occurs in the alternative of H1 for two by two matrices of attributes. Because the aggregate WML of AT search is greater than AL search in this case the alternative hypothesis is that for two by two matrices of attributes the AL order will yield a weakly larger relative frequency of correct choices (rather than the same relative frequency). Under these alternative hypotheses the results are the same as those presented in the results section: both hypotheses are rejected in favor of their alternatives.

References

1. Cowan N (2001) The magical number 4 in short-term memory: A reconsideration of mental storage capacity. Behavioral and Brain Sciences 24: 87–114.

2. Sanjurjo A (2014b) The role of memory load in search and choice. Working paper.
