# Supplementary material for: Search, Memory, and Choice Error: An Experiment
Source: PLoS One. 2015 Jun 29;10(6):e0126508. doi: 10.1371/journal.pone.0126508 (PMC4487248; doi:10.1371/journal.pone.0126508)
Supplement: S5 Appendix — (PDF) [file pone.0126508.s005.pdf]

Appendix S5: Brief review of multi-attribute search theory literature

The optimal sequential search *policy*, which characterizes both the optimal order of search and optimal stopping, has been characterized analytically when one of multiple attributes can be searched in each alternative, and alternatives are searched in a fixed order, with full recall [1], when there is one searchable alternative with two searchable attributes [2], and numerically for a multiple alternative, two attribute setting in which there is no recall, and the order of search across alternatives and attributes is fixed [3]. The optimal *order* of sequential search has been characterized in the case of a single searchable many-attribute alternative [2], or two [4], and the partial optimal order in the case of many attributes, many alternatives, no order restrictions, and full recall [4].

References

1. Neeman Z (1995) On determining the importance of attributes with a stopping problem. Mathematical Social Sciences 29: 195–212.
2. Klabjan D, Olszewski W, Wolinsky A (forthcoming) Attributes. Games and Economic Behavior.
3. Lim C, Bearden JN, Smith JC (2006) Sequential search with multiattribute options. Decision Analysis 3: 3–15.
4. Sanjurjo A (2014a) Search with multiple attributes: theory and empirics. Working paper.
